# Evaluation of intravascular contrast media transit times in coronary computed tomography angiography

**DOI:** 10.1590/0100-3984.2021.0063

**Published:** 2022

**Authors:** Raquel Rodrigues Borges, Tiago Nóbrega Morato, Alexandre Sérgio de Araujo Bezerra, Bruna Arrais Dias, Juliana Cavalcanti de Freitas Reinaux, Guilherme Urpia Monte, Luciano Farage

**Affiliations:** 1 Hospital Santa Marta, Brasília, DF, Brazil.; 2 Radiolinea Centro de Imagens, Brasília, DF, Brazil.; 3 Universidade de Brasília (UnB), Brasília, DF, Brazil.; 4 Instituto de Cardiologia do Distrito Federal (ICDF), Brasília, DF, Brazil.; 5 Centro Universitário Euroamericano (Unieuro), Brasília, DF, Brazil.

**Keywords:** Cardiac output, Contrast media, Computed tomography angiography

## Abstract

**Objective:**

To measure the transit times (TTs) of contrast agents among the injection site (antecubital vein), superior vena cava, pulmonary trunk, and ascending aorta, in coronary computed tomography angiography (CTA) examinations of outpatients with no history of cardiovascular or lung disease, thus defining reference values for those TTs.

**Materials and Methods:**

The contrast TTs from the injection site (antecubital vein) to the superior vena cava, from the superior vena cava to the pulmonary trunk, and from the pulmonary trunk to the ascending aorta were measured by monitoring contrast enhancement in real time (bolus tracking). Cardiac output was measured by the geometric method during the CTA examination and was correlated with the contrast TT.

**Results:**

Forty-three individuals were analyzed. The mean contrast TT was 13.1 s overall (from the antecubital vein to the ascending aorta), 3.0 s from the superior vena cava to the pulmonary trunk, and 7.2 s from the pulmonary trunk to the ascending aorta. There was a tendency toward a correlation between contrast TT and cardiac output (*p* = 0.055).

**Conclusion:**

The reference values established here for contrast TTs among the superior vena cava, pulmonary trunk, and ascending aorta will serve as a basis for clinical evaluation.

## Abstract (pt)

**Objetivo:**

Mensurar os tempos de trânsito de contraste (TTCs) entre o sítio de injeção em veia antecubital e a veia cava superior, tronco arterial pulmonar e aorta ascendente em exames de tomografia computadorizada de artérias coronárias de pacientes sem história de doenças cardiovasculares ou pulmonares, definindo padrões de normalidade para esses tempos de circulação.

**Materiais e Métodos:**

Os TTCs entre o sítio de injeção e a veia cava superior, tronco arterial pulmonar e aorta ascendente foram medidos com base nas imagens de monitoração (bolus tracking). O débito cardíaco foi calculado com base nas imagens de angiotomografia computadorizada pelo método geométrico e correlacionado com os TTCs.

**Resultados:**

Foram analisados 43 pacientes. O TTC médio entre o tronco arterial pulmonar e a aorta ascendente foi de 7,2 s, entre a veia cava superior e o tronco arterial pulmonar foi de 3 s e entre a veia antecubital e a aorta ascendente foi de 13 s. Houve tendência a correlação entre o TTC e o débito cardíaco, com valor de *p* de 0,055.

**Conclusão:**

Os valores de normalidade do TTC entre a veia cava superior, tronco arterial pulmonar e aorta ascendente foram estabelecidos, servindo de base para avaliação clínica.

## INTRODUCTION

Computed tomography (CT) of the chest with intravenous injection of iodinated contrast medium is widely used for the evaluation of individuals with cardiac or respiratory diseases. Due to its high spatial resolution, it allows detailed morphological evaluation. However, despite its good temporal resolution, CT has been underused for the assessment of functional parameters, which in the most diverse clinical situations provide information that is fundamental for the treatment decision-making process.

One functional parameter that can be studied by CT is cardiac function. The image acquisition can be synchronized with the electrocardiogram, allowing the subsequent processing of images of the heart in systole and diastole. Through the use of a well-established approach known as Simpson’s method^(1)^, CT allows the assessment of biventricular systolic and diastolic volumes, thereby allowing the ventricular ejection fraction and cardiac output to be calculated^(2,3)^. This technique is known as geometric analysis of cardiac function. However, the evaluation synchronized with the electrocardiogram implies the use of a higher radiation dose, a longer acquisition time, and a greater number of images for post-processing, increasing the evaluation time for the radiologist. It also requires that the post-processing and analysis of the images be performed with dedicated software.

Currently, cardiac magnetic resonance imaging (MRI) is the technique with the greatest reproducibility for the noninvasive assessment of ventricular volumes and the ejection fraction^(4,5)^. As in CT, volumes are measured with Simpson’s method^(6)^, and the correlation between CT and MRI is considered very good.

Another means of assessing cardiac function, widely used since the beginning of the 20th century, is the determination of cardiac output by constructing a curve of the concentration of a marker^(7)^. The method is based on detecting such a curve at a point away from the injection site. Iodinated contrast can be used as a marker, and CT density measurements show a linear relationship with the concentration of contrast in the blood^(8-10)^. Therefore, dynamic acquisition at the level of the large vessels allows the serial measurement of contrast concentrations, with subsequent estimation of cardiac output through the use of the Stewart-Hamilton equation. Conceptually, the technique is similar to the measurement of cardiac output using the Fick method.

A simplified form of assessment of overall cardiac function is to observe the time between injection of the contrast agent and its arrival in thoracic vascular structures. The assumption is that the time taken for the contrast agent to circulate is directly related to the cardiac output and the extent of the vascular territory to be covered, which is, in turn, proportional to the weight and height of the individual^(11-15)^. Experimental mathematical models have demonstrated that changes in cardiac output result in variations in the time from the injection of contrast to its arrival in the pulmonary trunk and ascending aorta^(12,13)^.

Vanhoenacker et al.^(16)^ suggested a simplified way to estimate ventricular function by determining the right-to-left ventricular contrast transit time (TT), which is a proxy for the contrast TT in the pulmonary circulation. The results obtained were promising and correlated well with the measurement of cardiac function by MRI. The use of this technique is attractive because it is simple, correlates with cardiac output determined by MRI, and does not require specific software for the analysis or for calibrating the scanner. However, one disadvantage of the technique proposed by the authors is the position at which the measurement is made. In clinical practice, the arrival of the contrast agent is not evaluated at the level of the ventricles but rather at the level of the pulmonary trunk, which limits the routine use of the method proposed by those authors.

Using the same theoretical reasoning as Vanhoenacker et al.^(16)^ and Shors et al.^(17)^, which allows the estimation of cardiac function, we hypothesized that it would be possible to estimate the time of peripheral venous circulation of a contrast medium, measured from the site of intravenous injection to its arrival in the superior vena cava, as well as its pulmonary vascular circulation time, by measuring the contrast TT from the pulmonary trunk to the ascending aorta. The use of this technique would be advantageous due to its simplicity in the acquisition of circulation times, not requiring a CT sequence with a high dose of radiation or calibration of the scanner. In this case, the images employed are acquired in the initial phase of the examination; that is, the phase of monitoring the arrival of the contrast medium at the target vessel (bolus tracking).

In addition to being directly related to cardiac output, the contrast TTs from the injection site to the superior vena cava and from the superior vena cava to the pulmonary trunk could be related to changes in pulmonary vascular resistance, and those TTs have been actively studied in patients with pulmonary hypertension^(18-20)^.

There have been studies showing that the contrast TT, measured from the injection site to the pulmonary trunk, correlates with right ventricular dysfunction, suggesting that the method can be used as an adjunct in the assessment of pulmonary hypertension, such as in retrospective studies of patients with confirmed pulmonary hypertension. In dynamic MRI studies of the pulmonary circulation in patients with pulmonary hypertension, Davarpanah et al.^(18)^ and Swift et al.^(21)^ also evaluated the contrast TT from the pulmonary trunk to the left atrium, finding a significant difference in the contrast TT between the patients that had a fatal outcome at follow-up and those that did not, demonstrating the potential for the use of the method in the assessment of pulmonary hypertension^(22)^. However, there are no data in the literature regarding the normal values for contrast TTs in the peripheral and pulmonary circulation. That makes it impossible to use the technique on a large scale.

In this study, we sought to measure contrast TTs among the injection site (antecubital vein), superior vena cava, pulmonary trunk, and ascending aorta in individuals undergoing contrast-enhanced coronary CT angiography (CTA). We also attempted to establish reference values for those TTs, the cardiac output of the individuals being calculated by Simpson’s method (volumetric quantification).

## MATERIALS AND METHODS

This was a retrospective study of a group of adult outpatients undergoing coronary CTA between December 2017 and April 2019. The subjects were recruited from among patients who were being followed for conditions unrelated to the focus of this study. The study was approved by the Research Ethics Committee of the Hospital Santa Marta, in the Federal District of Brasília, Brazil.

We included only individuals ≥ 18 years of age. Those with a history of heart failure, valvular heart disease, or lung disease were excluded, as were those in whom there were contraindications to the use of a contrast agent, as well as those with an ejection fraction lower than 55% and those with body surface area-indexed left ventricular end-diastolic and end-systolic volumes greater than 100 mL/m^2^ and 50 mL/m^2^, respectively.

### Image acquisition

The examinations were performed in a multidetector (64-slice) CT scanner (Brilliance; Philips Medical Systems, Best, The Netherlands). The images used in order to calculate the peripheral and pulmonary circulation times were obtained according to the standard examination protocol, with no increase in the volume of contrast medium injected or in the radiation dose. The non-ionic iodinated contrast medium ioversol (Optiray 350; Mallinckrodt, St. Louis, MO, USA) was administered via the antecubital vein of the right arm, through an 18-G catheter, at an infusion rate of 4 mL/s, followed by 30 mL of saline, also administered at 4 mL/s. The volume administered was calculated on the basis of the body mass of the individual (1.5 mL/kg).

**Figure 1:**
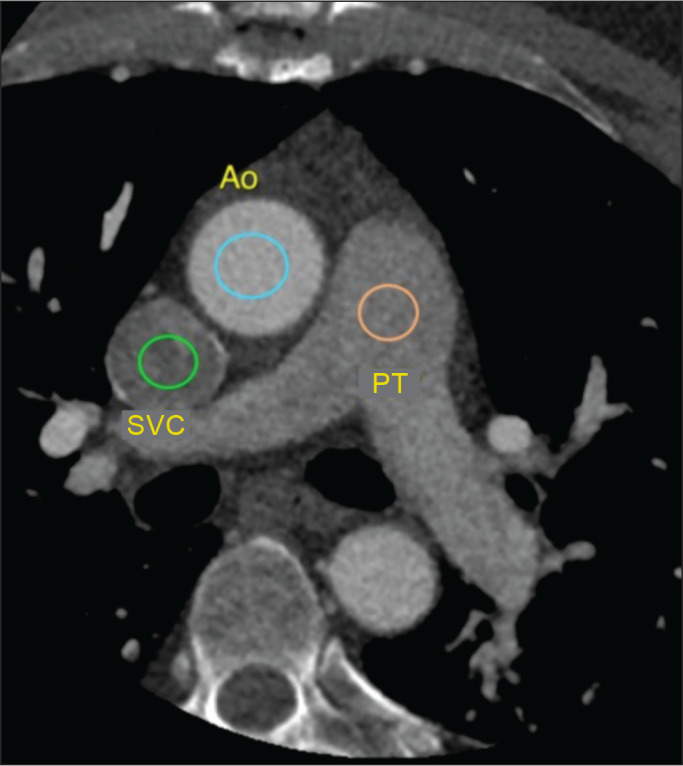
Attenuation monitoring sites (ROIs) within the superior vena cava (SVC), pulmonary trunk (PT), and ascending aorta (Ao).

We employed the technique known as bolus tracking, in which a region of interest (ROI) was drawn on the descending aorta, at the level of the pulmonary trunk, and the attenuation within the target vessel was measured once every second. Images of the coronary artery were acquired automatically when the attenuation within the vessel reached the threshold of 150 Hounsfield units (HU). The images acquired every second to monitor attenuation in the descending aorta were reconstructed in a new series to analyze the arrival times of the contrast agent in the superior vena cava, pulmonary trunk, and ascending aorta, all of which were visible in the slice used in order to monitor the descending aorta (Figure 1).

### Image analysis

The images employed for the calculation of vascular TTs were reconstructed at 1-s intervals. The resulting series was processed on a dedicated workstation (Philips Intellispace Portal; Philips Medical Systems) to calculate the circulation times.

### Peripheral venous circulation time

The peripheral venous circulation time was defined as the time from the start of intravenous injection of the contrast agent (into the antecubital vein) to the detection of increased attenuation related to the arrival of the contrast agent in the ROI drawn on the distal portion of the superior vena cava. Increased attenuation was defined as a ≥ 50 HU increase in the attenuation of the adjacent blood, in relation to that seen on an unenhanced image.

### Contrast TT from the superior vena cava to the pulmonary trunk

The contrast TT from the superior vena cava to the pulmonary trunk was defined as the time from the detection of an increase in attenuation related to the arrival of contrast agent in the ROI drawn on the superior vena cava to the detection of increase in attenuation related to the arrival of contrast agent in the ROI drawn on the pulmonary trunk. Increased attenuation was defined as a ≥ 50 HU increase in the attenuation of the adjacent blood, in relation to that seen on an unenhanced image.

### Contrast TT from the pulmonary trunk to the ascending aorta

The contrast TT from the pulmonary trunk to the ascending aorta was defined as the time from the detection of an increase in attenuation related to the arrival of contrast agent in the ROI drawn on the pulmonary trunk to the detection of increase in attenuation related to the arrival of contrast agent in the ROI drawn on the ascending aorta. Increased attenuation was defined as a ≥ 50 HU increase in the attenuation of the adjacent blood, in relation to that seen on an unenhanced image.

### Geometric analysis

For the geometric analysis, the contrast-enhanced CTA series was reconstructed in ten phases throughout the cardiac cycle. End-systolic, end-diastolic, and stroke volumes, as well as the ejection fraction, cardiac output, and cardiac index, were calculated from the contrast-enhanced images in a semi-automated manner on the dedicated workstation.

### Statistical analysis

To assess the distribution of normality of the contrast TTs, the D’Agostino-Pearson test was applied. The correlations between contrast TT values and cardiac output, as well as between contrast TT values and the cardiac index, were quantified by calculating Pearson’s correlation coefficient. All statistical tests were performed with GraphPad Prism software, version 7.0 (GraphPad Software Inc., San Diego, CA, USA).

## RESULTS

Forty-three individuals were included in the study. The main reason for referral was screening for coronary artery disease. The mean age was 56,2 years (range, 30-78 years), and 22 (51.2%) of the individuals were male. Table 1 details the main demographic data and heart rate data of the study sample. The radiation dose ranged from 30 mSv to 48 mSv, depending on the biotype of the individual.

**Table 1:** Demographic and clinical characteristics of the individuals evaluated.

Characteristic	(N = 43)
Age (years), mean ± SD	56.2 ± 12.9
Weight (kg), mean ± SD	76.2 ± 15.7
Height (cm), mean ± SD	163.7 ± 7.3
Heart rate (bpm), mean ± SD	59.0 ± 5.4
Male, n (%)	22 (51.2%)

SD, standard deviation; bpm, beats per minute.

The mean ejection fraction, estimated by the geometric method, was 63.0 ± 5.9% (range, 56.0-77.0%). The mean cardiac index was 2.6 ± 0.46 L/min/m^2^ (range, 2.0-3.9 L/min/m^2^). The results of the geometric analysis are shown in Table 2.

**Table 2:** Results of the geometric analysis.

Variable	Mean ± SD
End-diastolic volume*, mL/m^2^	69 ± 12.8
End-systolic volume*, mL/m^2^	25.7 ± 7.1
Stroke volume*, mL/m^2^	44 ± 7.7
Ejection fraction, %	63 ± 5.9
Myocardial mass, g	112 ± 31
Cardiac index, L/min/m^2^	2.6 ± 0.461

* Indexed to body surface area.

The mean time between peripheral injection of the contrast agent and its arrival in the superior vena cava was 3.0 ± 2.4 s, and the mean contrast TT from the superior vena cava to the pulmonary trunk was 2.9 ± 1.4 s. The mean contrast TT from the pulmonary trunk to the ascending aorta was 7.2 ± 2.2 s. Therefore, the mean time from peripheral injection of the contrast agent to its arrival in the ascending aorta was 13.1 ± 2.56 s.

**Figure 2:**
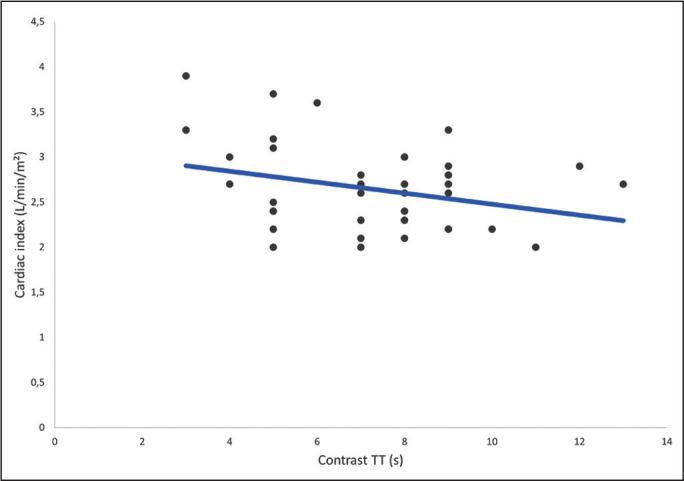
Dot plot of the correlation between contrast TT and cardiac index (r = -0.29).

The D’Agostino-Pearson test showed that, for the contrast TT from the pulmonary trunk to the ascending aorta, the data distribution was normal. There was a trend toward a significant correlation between the cardiac index and the contrast TT from the pulmonary trunk to the ascending aorta (*p* = 0.055). That contrast TT showed statistically significant correlations with the heart rate, left ventricular ejection fraction, and stroke volume (Table 3). As depicted in Figure 2, the contrast TT showed a significant inverse correlation with the cardiac index (r = -0.29).

**Table 3:** Correlations that the contrast TT from the pulmonary trunk to the ascending aorta showed with other parameters.

Comparison	r	P
PT-AA × HR	0.3621	0.017
PT-AA × LVEF	-0.3686	0.015
PT-AA × SV	-0.3027	0.048
PT-AA × CI	-0.2938	0.055

PT, pulmonary trunk; AA, ascending aorta; PT-AA, contrast TT from the PT to the AA; HR, heart rate; LVEF, left ventricular ejection fraction; SV, stroke volume; CI, cardiac index.

## DISCUSSION

Investigation of the TTs of markers has long been used as a form of cardiovascular assessment. At the end of the 20th century and in the first decades of the 21st, there were numerous studies employing modern imaging methods, primarily MRI, CT, and ultrasound, for the investigation of circulatory TTs^(8,10,17,18,23-25)^.

The assessment of contrast TTs in bolus tracking studies is directly related to cardiac function. The method does not require an additional injection of contrast medium, nor an extra dose of radiation. In addition, post-processing is minimal, requiring only a few seconds to count, in the source images, the time it takes for the contrast medium to circulate between two structures of interest. Davarpanah et al.^(18)^ investigated the contrast TT from the injection site to the pulmonary trunk in patients with pulmonary hypertension. The authors detected a significant difference between the contrast TT observed for the patients with pulmonary hypertension (defined as an estimated right ventricular systolic pressure greater than 40 mmHg) and that observed for the control patients. The contrast TT also differed significantly between the patients with signs of right ventricular dysfunction on echocardiography and those without. In addition to the diagnostic value of the assessment of contrast TTs, the study of contrast medium dynamics allows image acquisition times to be predicted more consistently^(11-14,24)^.

Although there have been some studies on the subject of the assessment of contrast TTs in populations of patients with pulmonary hypertension and heart failure, a review of the literature in the indexing databases revealed no population-based studies aimed at establishing reference values for contrast TT. In view of that gap, the aim of this study was to determine a population-based reference value for the contrast TT from the pulmonary trunk to the ascending aorta, because it most reliably simulates the positioning of the slice used for monitoring in various types of thoracic CTA examinations, including CTA of the pulmonary arteries, coronary arteries, and aorta. That makes the measurement of contrast TTs directly applicable in clinical practice. No significant difference is expected in relation to the measures described in previous studies, given the equidistance of the observation points proposed by our group and those used by other groups^(17,18,22)^. In the present study, the mean contrast TT from the pulmonary trunk to the ascending aorta was 7.2 s, being comparable to that obtained for control groups in studies that performed the same measurement, as in the study conducted by Davarpanah et al.^(18)^, in which the mean control group contrast TT was 6.6 s, and in the study conducted by Skrok et al.^(22)^, in which it was approximately 6.4 s.

One of the current concerns of radiological practice is the radiation dose, which is why one of the strengths of the technique proposed here is that it does not call for a significantly higher dose of radiation than that used routinely. The use of monitoring from the initiation of contrast agent injection allowed the acquisition of approximately 60 additional chest images, at a low current and voltage (typically 100 kVp and 50 mAs, respectively). Some vascular studies, such as CTA of the aorta, can be performed without monitoring the arrival of the contrast medium in the target structure from the initiation of the injection. In such studies, the CT scanner is usually programmed to start monitoring only after a fixed interval, typically 10-15 s, which minimizes the radiation dose but precludes the analysis of TTs. The estimated additional radiation dose for the monitoring proposed is approximately 0.07 mSv, which can be considered very low (the mean annual exposure in the general population is on the order of 2 mSv).

Most studies of contrast TTs have used the right-to-left ventricular contrast TT as a reference^(9,10,16-18)^. To make that measurement, it is necessary to perform an additional sequence, with the use of a test injection of contrast medium or modification of the standard protocol for the acquisition of CTA images.

The size of our sample was sufficient to study the mean contrast TTs, with normal data distribution for the contrast TT from the pulmonary trunk to the ascending aorta. Therefore, these findings could be reproduced in larger samples of patients.

As previously stated, the contrast TT showed a significant inverse correlation with cardiac output. The low r value in our study is probably related to the selection of individuals without cardiovascular alterations and to the relatively small size of our sample. The initial objective of the work was not to prove that correlation, which has previously been demonstrated in studies of contrast medium dynamics and in thermodilution studies^(7,9-11)^, but rather to establish the mean value of normality for the contrast TT in question. We believe that once a reference value for contrast TT has been established, it could be used as a screening criterion. The identification of individuals with altered contrast TTs merits clinical attention and complementation with specific diagnostic methods such as echocardiography and quantification of pro-B-type natriuretic peptide.

## CONCLUSION

We have established a reference value for the contrast TT from the pulmonary trunk to the ascending aorta, which can serve as a basis for clinical evaluation. The use of this technique could make radiologists more confident in suggesting or excluding heart disease in patients referred for major CT applications.

## References

[1] Thiele H, Paetsch I, Schnackenburg B (2002). Improved accuracy of quantitative assessment of left ventricular
volume and ejection fraction by geometric models with steady-state free
precession. J Cardiovasc Magn Reson.

[2] Coche E, Vlassenbroek A, Roelants V (2005). Evaluation of biventricular ejection fraction with ECG-gated
16-slice CT: preliminary findings in acute pulmonary embolism in comparison
with radionuclide ventriculography. Eur Radiol.

[3] Scheffel H, Stolzmann P, Leschka S (2012). Ventricular short-axis measurements in patients with pulmonary
embolism: effect of ECG-gating on variability, accuracy, and risk
prediction. Eur J Radiol.

[4] Grothues F, Braun-Dullaeus R (2009). Serial assessment of ventricular morphology and
function. Heart Fail Clin.

[5] Hundley WG, Bluemke DA, Finn JP, American College of Cardiology Foundation Task Force on Expert
Consensus Documents (2010). ACCF/ACR/AHA/NASCI/SCMR 2010 expert consensus document on
cardiovascular magnetic resonance: a report of the American College of
Cardiology Foundation Task Force on Expert Consensus
Documents. J Am Coll Cardiol.

[6] Raman SV, Shah M, McCarthy B (2006). Multi-detector row cardiac computed tomography accurately
quantifies right and left ventricular size and function compared with
cardiac magnetic resonance. Am Heart J.

[7] Morris LE, Blumgart HL (1957). Velocity of blood flow in health and disease. Circulation.

[8] Garrett JS, Lanzer P, Jaschke W (1985). Measurement of cardiac output by cine computed
tomography. Am J Cardiol.

[9] Ludman PF, Coats AJ, Poole-Wilson PA (1993). Measurement accuracy of cardiac output in humans:
indicator-dilution technique versus geometric analysis by ultrafast computed
tomography. J Am Coll Cardiol.

[10] Mahnken AH, Klotz E, Hennemuth A (2003). Measurement of cardiac output from a test-bolus injection in
multislice computed tomography. Eur Radiol.

[11] Bae KT (2003). Peak contrast enhancement in CT and MR angiography: when does it
occur and why? Pharmacokinetic study in a porcine model. Radiology.

[12] Bae KT (2010). Optimization of contrast enhancement in thoracic
MDCT. Radiol Clin North Am.

[13] Bae KT, Heiken JP, Brink JA (1998). Aortic and hepatic contrast medium enhancement at CT. Part I.
Prediction with a computer model. Radiology.

[14] Bae KT, Heiken JP, Brink JA (1998). Aortic and hepatic contrast medium enhancement at CT. Part II.
Effect of reduced cardiac output in a porcine model. Radiology.

[15] Bae KT, Tran HQ, Heiken JP (2000). Multiphasic injection method for uniform prolonged vascular
enhancement at CT angiography: pharmacokinetic analysis and experimental
porcine model. Radiology.

[16] Vanhoenacker PK, Van Hoe LR (2007). A simple method to estimate cardiac function during routine
multi-row detector CT exams. Eur Radiol.

[17] Shors SM, Cotts WG, Pavlovic-Surjancev B (2003). Heart failure: evaluation of cardiopulmonary transit times with
time-resolved MR angiography. Radiology.

[18] Davarpanah AH, Hodnett PA, Farrelly CT (2011). MDCT bolus tracking data as an adjunct for predicting the
diagnosis of pulmonary hypertension and concomitant right-heart
failure. AJR Am J Roentgenol.

[19] Swift AJ, Telfer A, Rajaram S (2014). Dynamic contrast-enhanced magnetic resonance imaging in patients
with pulmonary arterial hypertension. Pulm Circ.

[20] Swift AJ, Wild JM, Nagle SK (2014). Quantitative magnetic resonance imaging of pulmonary
hypertension: a practical approach to the current state of the
art. J Thorac Imaging.

[21] Swift AJ, Rajaram S, Hurdman J (2013). Noninvasive estimation of PA pressure, flow, resistance with CMR
imaging: derivation and prospective validation study from the ASPIRE
registry. JACC Cardiovasc Imaging.

[22] Skrok J, Shehata ML, Mathai S (2012). Pulmonary arterial hypertension: MR imaging-derived first-pass
bolus kinetic parameters are biomarkers for pulmonary hemodynamics, cardiac
function, and ventricular remodeling. Radiology.

[23] Müller HM, Rehak PH, Puchinger M (2009). Measurement of cardiac output and pulmonary transit time for
assessment of pulmonary vascular resistance in domestic
piglets. Exp Physiol.

[24] Francois CJ, Shors SM, Bonow RO (2003). Analysis of cardiopulmonary transit times at contrast
material-enhanced MR imaging in patients with heart disease. Radiology.

[25] Choi BG, Sanai R, Yang B (2014). Estimation of cardiac output and pulmonary vascular resistance by
contrast echocardiography transit time measurement: a prospective pilot
study. Cardiovasc Ultrasound.

